# Arteriovenous malformation in pancreas mimicking hypervascular tumor

**DOI:** 10.1002/jgh3.12343

**Published:** 2020-04-18

**Authors:** Keisuke Ishigami, Tomoya Sakuma, Masato Saito, Yujiro Kawakami, Yoshiharu Masaki, Ayako Murota, Masayo Motoya, Yasutoshi Kimura, Hiroshi Nakase

**Affiliations:** ^1^ Department of Gastroenterology and Hepatology Sapporo Medical University School of Medicine Sapporo Japan; ^2^ Sapporo Medical University School of Medicine Sapporo Japan; ^3^ Department of Radiology Oncology Sapporo Medical University School of Medicine Sapporo Japan; ^4^ Department of Surgery, Surgical Oncology and Science Sapporo Medical University School of Medicine Sapporo Japan

**Keywords:** arteriovenous malformation, pancreas, pseudo tumor

## Abstract

Arteriovenous malformation (AVM) is defined as a disease that causes blood flow abnormality due to anastomoses of the arteries and veins. AVM can occur in any gastrointestinal tract, but pancreatic AVM (P‐AVM) is very rare. Previous reports demonstrated that contrast‐enhanced CT (CECT) typically showed abnormal vascular network in pancreas. We present a 58‐year old man with a history of acute pancreatitis. He was referred to our hospital for examination of pancreatic mass. CECT showed a round‐shaped hypervascular lesion with a diameter of 8 mm in the head of the pancreas. Selective angiography showed vascular network and early visualization of superior mesenteric vein. We finally diagnosed this case as P‐AVM. He underwent duodenum preserving pancreatic head resection. Histological findings confirmed the preoperative diagnosis of P‐AVM.

## Graphical abstract

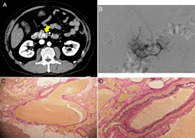

An asymptomatic 58‐year old man with a history of acute pancreatitis was referred to our hospital for examination of a round‐shaped hypervascular lesion with a diameter of 8 mm in the head of the pancreas. Selective angiography showed vascular network in the pancreatic head and super‐selective injections from pancreaticoduodenal artery revealed early visualization of superior mesenteric vein. Pancreatic arteriovenoous malformation typically showed multiple discrete intrapancreatic vessels, and few cases were reported as hypervascular tumorous lesion.

## Clinical history

**Figure 1:**
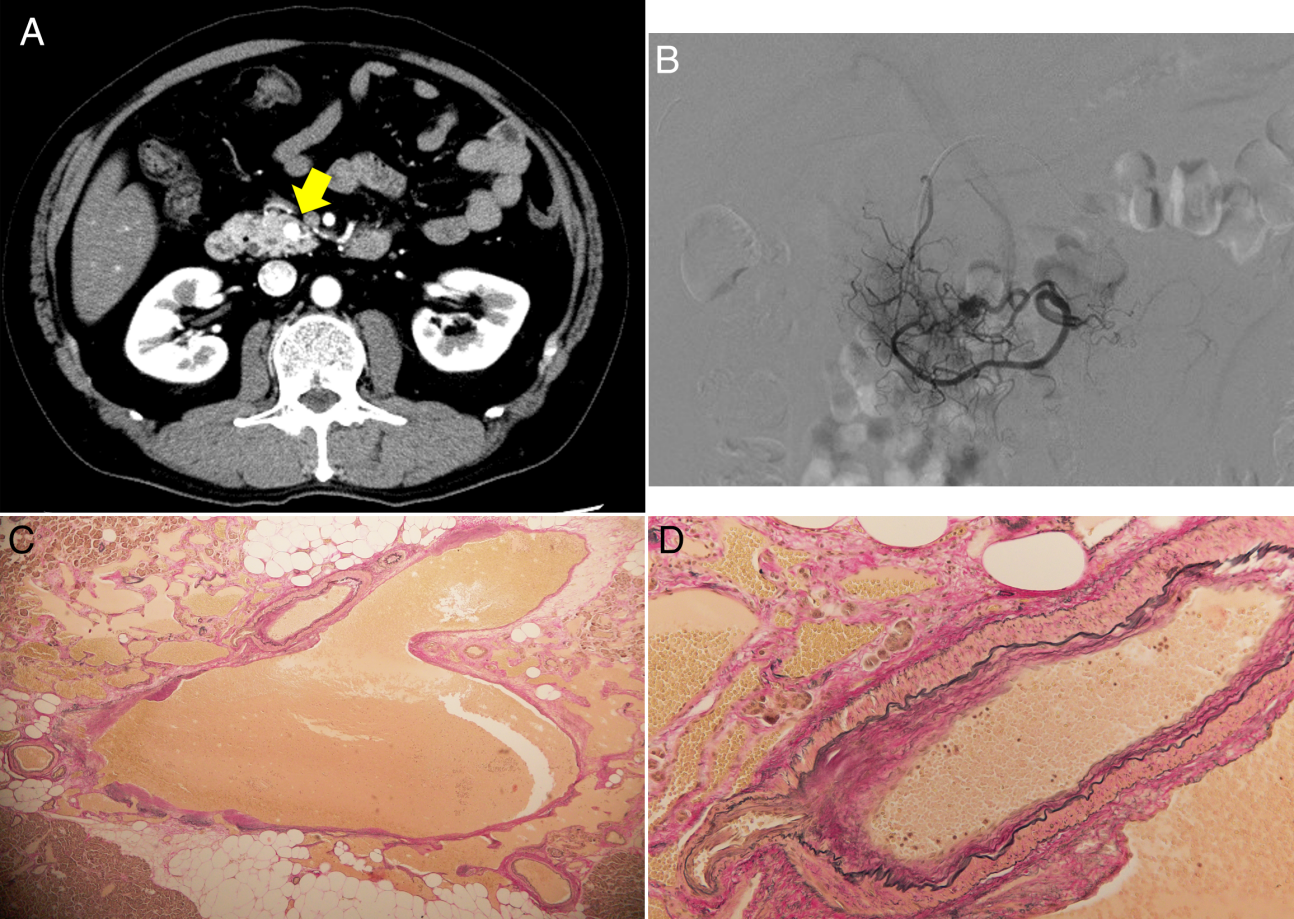
(a) Contrast‐enhanced CT revealed a hypervascular tumorous lesion in the head of the pancreas (arrow). (b) Selective angiography showed abnormal vascular network and early visualization of superior mesenteric vein. (c) Elastica van Gieson (EVG) staining (×40) showed increased abnormal blood vessels. (d) EVG staining (×200) focused on an anastomosis of a vein and a small artery with inappropriate tunica media.

An asymptomatic 58‐year‐old man with a history of acute pancreatitis was referred to our hospital for examination of pancreatic mass. Physical examination revealed a soft abdomen without any tenderness. Contrast‐enhanced computed tomography showed a round‐shaped hypervascular lesion with a diameter of 8 mm in the head of the pancreas (Fig. 1a). Pancreatic enzyme levels were not elevated. The serum levels of tumor markers were as follows: carcinoembryonic antigen (CEA), 5.0 ng/mL; carbohydrate antigen 19‐9 (CA19‐9), <2.0 ng/mL; neuron‐specific enolase, 13.9 ng/mL; and progastrin‐releasing peptide, 52.1 pg/mL. Serum levels of gastrin and insulin were within normal range. We could not detect the pancreatic mass on B‐mode ultrasonography, but color‐doppler ultrasonography showed a mosaic pattern of blood flow in accordance with pancreatic mass. ^111^In‐pentetreotide somatostatin receptor (SSTR) scintigram failed to detect any positive accumulation in the pancreas. Selective angiography showed a vascular network in the pancreatic head, and super‐selective injections from the pancreaticoduodenal artery revealed early visualization of superior mesenteric vein (Fig. 1b).

Based on these findings, we diagnosed this case as pancreatic arteriovenous malformation (P‐AVM). He underwent duodenum‐preserving pancreatic head resection because we could not exclude the relationship between P‐AVM and a past history of pancreatitis. Histological findings revealed the anastomosis of abnormal arteries and veins, which confirmed the preoperative diagnosis of P‐AVM (Fig. 1c,d).

## Discussion

P‐AVM is defined as a disease that causes blood flow abnormality due to anastomoses of the arterial and portal systems in the pancreas.^1^ AVM can occur in any gastrointestinal (GI) tract, but P‐AVM is very rare.^2^ Abdominal pain and melena were major clinical symptoms that led to the diagnosis of P‐AVM.^3^, ^4^ Recently, cases of asymptomatic P‐AVM have been increasingly reported with technological advances. Contrast‐enhanced computed tomography typically shows multiple discrete intrapancreatic vessels, best demonstrated in early arterial phase. Interestingly, in the present case, P‐AVM presented regular round‐shaped mimicking pancreatic hypervascular tumor, such as neuroendocrine tumor. Surgical treatment is considered curative and is selected, especially in symptomatic case. Transcatheter arterial embolization is also performed; however, it is sometimes difficult to achieve complete embolization because P‐AVMs are typically supplied by multiple feeding arteries.^5^ We should keep P‐AVM in mind as one of differential diagnosis when we encounter hypervascular tumorous lesions in pancreas.
